# popDMS infers mutation effects from deep mutational scanning data

**DOI:** 10.1093/bioinformatics/btae499

**Published:** 2024-08-08

**Authors:** Zhenchen Hong, Kai S Shimagaki, John P Barton

**Affiliations:** Department of Physics and Astronomy, University of California, Riverside, CA 92521, United States; Department of Computational and Systems Biology, University of Pittsburgh School of Medicine, PA 15260, United States; Department of Physics and Astronomy, University of California, Riverside, CA 92521, United States; Department of Computational and Systems Biology, University of Pittsburgh School of Medicine, PA 15260, United States; Department of Physics and Astronomy, University of Pittsburgh, PA 15260, United States

## Abstract

**Summary:**

Deep mutational scanning (DMS) experiments provide a powerful method to measure the functional effects of genetic mutations at massive scales. However, the data generated from these experiments can be difficult to analyze, with significant variation between experimental replicates. To overcome this challenge, we developed popDMS, a computational method based on population genetics theory, to infer the functional effects of mutations from DMS data. Through extensive tests, we found that the functional effects of single mutations and epistasis inferred by popDMS are highly consistent across replicates, comparing favorably with existing methods. Our approach is flexible and can be widely applied to DMS data that includes multiple time points, multiple replicates, and different experimental conditions.

**Availability and implementation:**

popDMS is implemented in Python and Julia, and is freely available on GitHub at https://github.com/bartonlab/popDMS.

## 1 Introduction

Understanding the relationship between protein sequence and phenotype is a central question in evolution and protein engineering. In recent years, a new family of experimental methods, commonly referred to as deep mutational scanning (DMS) or multiplexed assays for variant effects (MAVEs), have been developed to measure the functional effects of large numbers of mutations simultaneously (Fowler *et al.* 2010, Gasperini *et al.* 2016). DMS experiments typically work by generating a vast library of protein variants that are then passed through rounds of selection that favor functional variants while eliminating deleterious ones (Fowler and Fields 2014). One can then compare variant frequencies in the pre- and post-selection libraries to estimate the functional effects of mutations. This approach has been successfully applied in a wide variety of contexts, from studying the function of enzymes (Romero *et al.* 2015) and tRNAs (Li *et al.* 2016) to measuring the mutational tolerance of influenza (Thyagarajan and Bloom 2014, Doud *et al.* 2018, Lee *et al.* 2018) and human immunodeficiency virus (HIV-1) (Haddox *et al.* 2016, Dingens *et al.* 2017, Haddox *et al.* 2018) surface proteins.

Despite the success of DMS experiments, popular approaches for analyzing DMS data yield modest correlations between the inferred functional effects of mutations in experimental replicates. Thus, a significant amount of variance in the data remains unexplained. Some methods use the ratios between post- and pre-selection variant frequencies, known as enrichment ratios, to estimate mutation effects (Fowler *et al.* 2011, Hietpas *et al.* 2011, Bloom 2015). Ratio-based methods may be sensitive to noise when variant counts are low, a common occurrence in DMS experiments. Methods based on regression (Araya *et al.* 2012, Starita *et al.* 2015, Matuszewski *et al.* 2016, Rich *et al.* 2016, Rubin *et al.* 2017) provide improved performance, but substantial uncertainty in the inferred effects of different mutations persists.

## 2 Results

We developed a method, popDMS, to estimate the functional effects of mutations in DMS experiments using statistical methods from population genetics (Supplementary Information). In our approach, we view rounds of phenotypic selection in experiments as analogous to rounds of reproduction in natural populations. We quantify the effect of each mutation *i* by a selection coefficient *s_i_*, which describes the relative advantage or disadvantage of the mutation for surviving selection in the experiment. For simplicity, we assume that the total fitness of a sequence with multiple mutations is the sum of the corresponding selection coefficients. We then use the Wright-Fisher (WF) model, an evolutionary model from population genetics, to quantify the likelihood of the experimentally observed variant frequencies over time as a function of the selection coefficients, $L({\left(z(t_{k})\right)}_{k=0}^{K}|s)$ (see Supplementary Information for details). The $z(t_{k})$ represent vectors of variant frequencies z at different times *t_k_*. The WF model defines the relationship between “fitness” and frequency change, and allows us to model competition between variants. We then use sequence data to estimate the effects of mutations on fitness in experiments.

To regularize our estimates, we introduce a Gaussian prior distribution $P_{\text{prior}}(s)$ for the selection coefficients. Leveraging recently developed computational methods (Sohail *et al.* 2021, 2022, Lee *et al.* 2022), we can identify the selection coefficients that represent the best compromise between fitting the data and minimizing the prior distribution,

(1) $$\hat{s}=\underset{s}{\arg\max}\;L\left(s|{\left(z(t_{k})\right)}_{k=0}^{K}\right)\;P_{\text{prior}}(s).$$

Typically, we adjust the width of the prior distribution based on the data, but a fixed value can also be specified (Supplementary Information). The Gaussian prior is equivalent to an *L*_2_-norm penalty on the selection coefficients, or ridge regression.

popDMS has several computational strengths. First, the use of regularization for the selection coefficients curbs the inference of strong functional effects in the absence of strong statistical evidence. Our likelihood framework further allows us to derive joint estimates of selection coefficients across replicates that are guided by levels of evidence in the data, rather than simply averaging the inferred functional effects of mutations across replicates. When information about sequencing error rates is available, we can perform error correction for variant frequencies.

In simulations, we found that popDMS was robust to sampling noise and provided stronger correlations between inferred variant effects across replicates than common methods based on enrichment ratios or regression (Supplementary Fig. S1). The variant effects inferred by popDMS were also more similar to true, underlying ones than alternative approaches, even with the addition of negative binomial sampling noise (Supplementary Fig. S2, see Supplementary Information).

**Figure 1.**
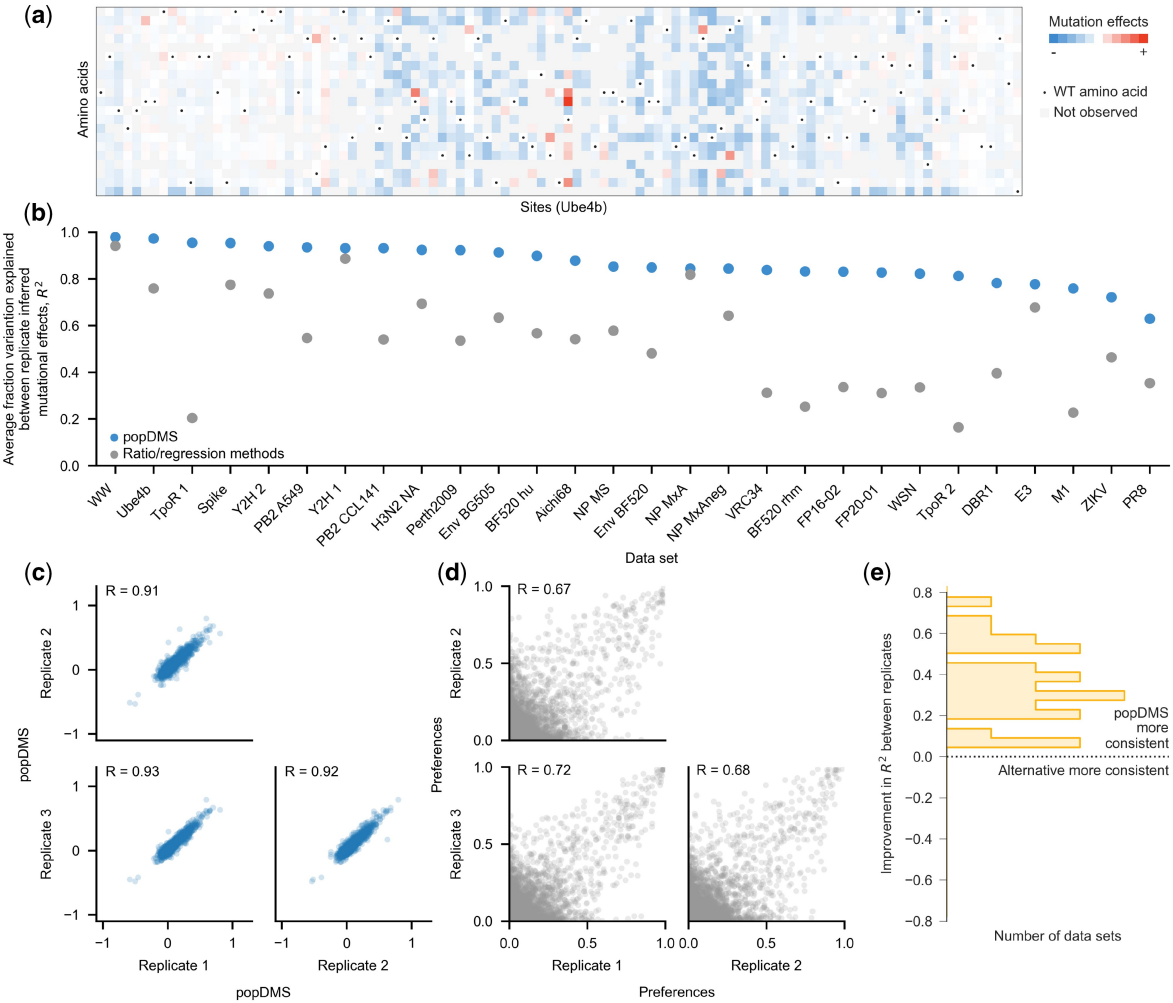
popDMS overview. (a) Example of the effects of mutations inferred by popDMS for the Ube4b protein (Starita *et al.* 2013). (b) Across 28 datasets, popDMS infers more consistent mutational effects than previous ratio/regression-based methods. To illustrate consistency between replicates, we show (c) selection coefficients inferred across replicates for the HIV-1 envelop BF520 dataset (Haddox *et al.* 2018), compared with (d) enrichment ratios for the same data. (e) popDMS gains in consistency across replicates are often substantial, improving *R*^2^ by an average of 0.34

Next, we analyzed a collection of 28 DMS datasets with popDMS (Araya *et al.* 2012, Starita *et al.* 2013, Findlay *et al.* 2014, Doud *et al.* 2015, Starita *et al.* 2015, Li *et al.* 2016, Ashenberg *et al.* 2017, Dingens *et al.* 2018, Haddox *et al.* 2018, Hom *et al.* 2019, Soh *et al.* 2019, Bridgford *et al.* 2020, Roop *et al.* 2020, Starr *et al.* 2020, Lei *et al.* 2023). These datasets were generated and analyzed using a variety of experimental techniques and analytical methods (see Supplementary Table S1). Like the functional metrics introduced by previous methods, selection coefficients provide an intuitive visualization of the functional effects of mutations (Fig. 1a). To quantify the consistency of different analytical methods, we computed the Pearson correlation *R* between mutation effects inferred from replicates of the same experiment. We found that mutation effects inferred by popDMS had higher correlations between replicates than those inferred by prior methods for all the datasets that we considered (Fig. 1b). The rank correlations between replicates were also typically higher for popDMS than for other approaches, showing that the consistency of the inferred mutational effects is not simply due to rescaling (Supplementary Fig. S3). Furthermore, our selection coefficients compared favorably with the frequencies of amino acid variants in influenza viruses in a natural population (Thyagarajan and Bloom 2014) (see Supplementary Information).

To illustrate performance in a typical case, we show selection coefficients inferred for mutations in the HIV-1 envelope protein BF520 (Fig. 1c) compared with enrichment ratios (Fig. 1d) for the same data (Haddox *et al.* 2018). Improvements in consistency across replicates with popDMS were often substantial. The mean improvement in *R*^2^ for variant effects was 0.35, with 6 out of 28 datasets showing an improvement in *R*^2^ of >0.50 (Fig. 1e).

In addition to the modified form of our estimator for variant effects, regularization also contributes to the improved correlation between replicates by shrinking effects with little support in the data toward zero (see Supplementary Fig. S4). As we discuss below, we also treat wild-type (WT) amino acids differently than most ratio- or regression-based approaches. Because WT residues are typically among the fittest at each site, changes to these terms can have particularly large effects on consistency between replicates.

**Figure 2.**
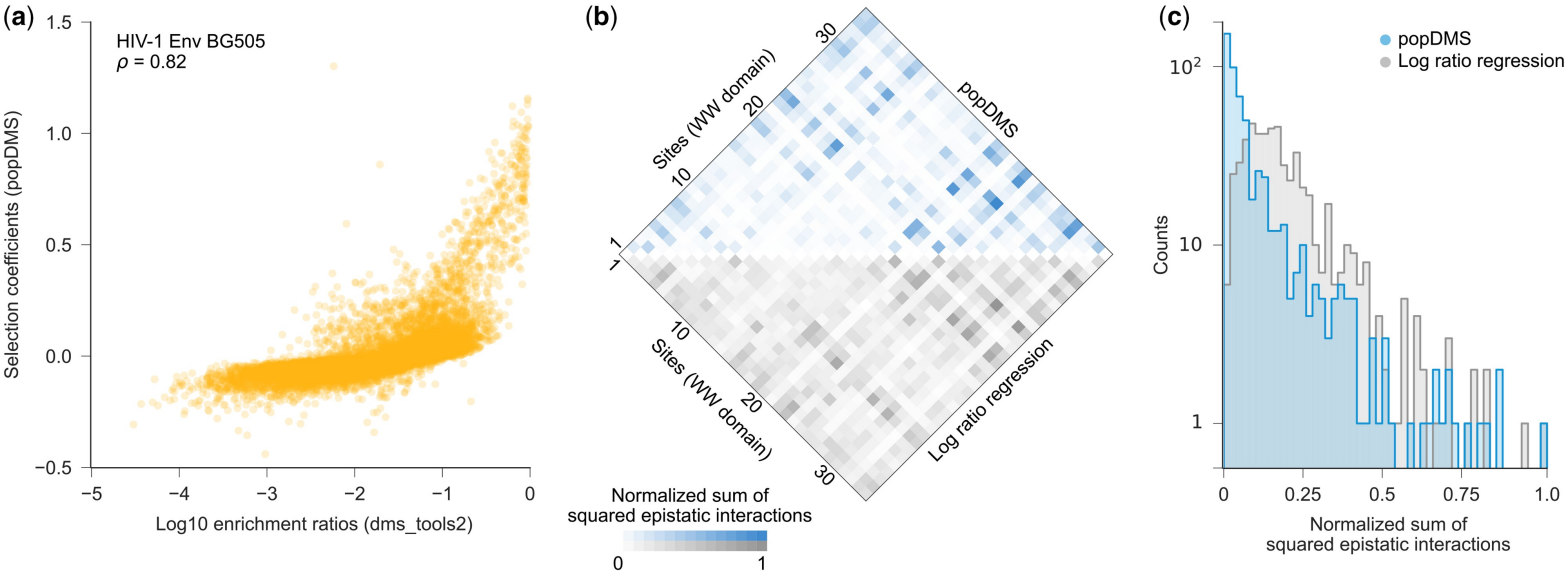
Mutation effects inferred by popDMS are broadly consistent with alternative methods. (a) For the HIV-1 Env BG505 dataset, selection coefficients inferred by popDMS are congruent with enrichment ratios computed using dms_tools2 (Spearman’s *ρ *= 0.84). At some sites, significant differences are observed (see Supplementary Fig. S5). (b) In the hYAP65 WW domain dataset, similar sites are inferred to have strong epistatic interactions using popDMS and log ratio regression (Araya *et al.* 2012). Interactions inferred in ref. (Araya *et al.* 2012) have been transformed to compare more directly with interactions inferred by popDMS, and both sets of interactions are normalized to scale between zero and one (Supplementary Information). (c) Epistatic interactions inferred by popDMS are substantially sparser than those inferred with the regression-based approach (Araya *et al.* 2012)

We then asked how similar the selection coefficients inferred by popDMS are to mutation effects inferred by previous methods. Across the experimental datasets that we tested, popDMS results were broadly consistent with existing metrics (average Pearson’s *R *=* *0.74). This correlation is similar to the average correlation between replicates of the same dataset using current ratio- or regression-based methods (average Pearson’s *R *=* *0.70). Figure 2a shows a typical example, comparing selection coefficients inferred by popDMS with enrichment ratios for the HIV-1 Env BG505 dataset (Dingens *et al.* 2018).

While the inferred mutation effects agreed for most sites, some showed qualitative differences (Supplementary Fig. S5). One factor underlying this result is that popDMS models variants with high initial frequencies, such as WT or reference amino acids, in the same way as other, low-frequency variants (see Supplementary Information). In alternative methods, the statistical treatment for WT amino acids is often different than for other variants.

Beyond inferring the effects of individual mutations, we can apply popDMS to estimate pairwise epistatic interactions between variants at different sites. We inferred epistatic interactions in an hYAP65 WW domain dataset using popDMS, which we also compared with previous results (Araya *et al.* 2012). Due to different conventions in defining epistasis, we transformed the functional measurements defined in ref. (Araya *et al.* 2012) to more directly compare with our results (Supplementary Information). To more clearly identify strongly interacting pairs of sites, we computed the sum of squared epistatic interactions between all pairs of amino acids at each pair of sites in the WW domain, using both popDMS and the previous regression-based approach. Our results showed good agreement with the pairs of sites that were previously inferred to have the strongest epistatic interactions (Fig. 2b). However, epistatic interactions inferred by popDMS were substantially sparser than those that had been inferred before (Fig. 2c). Given the enormous number of possible epistatic interactions between amino acid variants at different sites, sparsity is an attractive statistical feature that can facilitate focus on a smaller number of biologically important interactions.

## 3 Discussion

In summary, popDMS is an efficient, reliable approach for inferring mutation effects from DMS data, which is grounded in evolutionary theory. Across simulations and a wide array of datasets, we found that popDMS infers more consistent mutation effects than the popular alternatives used here. Our approach allows us to combine statistical power across multiple replicates, and it is also capable of inferring epistatic interactions given appropriate data. popDMS is written in Python3 and C++, and uses codon counts in dms_tools format (Bloom 2015) or sequence counts in MaveDB format (Esposito *et al.* 2019) as input, with code and example visualizations freely available on GitHub (https://github.com/bartonlab/popDMS, Supplementary Information).

Here, we have focused on the correlations of inferred mutational effects between experimental replicates to quantify the consistency of different inference methods. By this statistical measure, popDMS is more consistent on average than current ratio- and regression-based methods, including both correlations between values (Pearson correlations) and the ranks of mutational effects (Spearman correlations). We also found that selection coefficients inferred by popDMS more closely matched with underlying fitness parameters in simulations. However, greater biological relevance could only be established through experiments. Future studies that experimentally test the predictions of different inference methods would be of great interest.

## Supplementary Material

btae499_Supplementary_Data
