# Supplementary material for: popDMS infers mutation effects from deep mutational scanning data
Source: Bioinformatics. 2024 Aug 8;40(8):btae499. doi: 10.1093/bioinformatics/btae499 (PMC11335369; doi:10.1093/bioinformatics/btae499)
Supplement: btae499_Supplementary_Data [file btae499_supplementary_data.pdf]

Methods

Evolutionary model

We model rounds of selection in deep mutational scanning experiments like rounds of reproduction in an evolving population. For this purpose, we use the Wright-Fisher (WF) model[1], a simple model from population genetics where individuals in a population undergo discrete rounds of mutation, selection, and reproduction. We define the WF model as follows. We assume that the population consists of N individuals, each of which possesses a genetic sequence of length L. Each site in the genetic sequence can take on one of q possible states, resulting in $M = q^L$ possible genotypes.

In the context of DMS experiments, we are typically interested in the properties of proteins with different amino acid variants at each site, and thus we use $q = 21$ for data analyses (representing 20 amino acids and a stop, which could also be further extended to account for gaps). However, the framework that we consider is more general. One could consider nucleotide sequences with $q = 4$ states (A, C, T, G), $q = 64$ codons, and so forth.

At each time t, the state of the population is defined by a genotype frequency vector $\boldsymbol{z}(t) = (z_1(t), z_2(t), \ldots, z_M(t))$, where $z_a(t) = n_a(t)/N$, with $n_a(t)$ representing the number of individuals that have genotype a at time t. Under the WF model, the probability of observing genotype frequencies $\boldsymbol{z}(t+1)$ in the next generation is binomial,

$$P\left(\boldsymbol{z}(t+1) \mid \boldsymbol{z}(t)\right) = N! \prod_{a=1}^{M} \frac{p_a(\boldsymbol{z}(t))^{N z_a(t+1)}}{(N z_a(t+1))!}, \quad \textbf{(1)}$$

with

$$p_a(\boldsymbol{z}(t)) = \frac{z_a(t) f_a + \sum_{b \neq a} \left(\mu_{ba} z_b(t) - \mu_{ab} z_a(t)\right)}{\sum_{b=1}^{M} z_b(t) f_b}. \quad \textbf{(2)}$$

Here f_a is the *fitness* of genotype a, defined in detail below, and μ_{ab} is the probability of mutation from genotype a to genotype b in one generation. In typical experiments, mutation rates are low enough that we assume μ_{ab} is zero across all pairs of genotypes a, b.

We assume that the fitness of each genotype depends linearly on the amino acid (or nucleotide, codon, etc.) content of the sequence,

$$f_a = 1 + \sum_i s_i g_i^a. \quad \textbf{(3)}$$

In Eq. (3), the s_i are *selection coefficients* for each variant i, which quantify the effect of that variant on fitness. If s_i is positive, then the variant is beneficial, and if s_i is negative, then the variant is deleterious. Here g_i^a is an indicator variable, which is equal to one if genotype a possesses the variant i, and zero otherwise. The variant indicator i is a generic index that runs across all possible amino acids or states at each site in the sequence. For example, let us define a genotype sequence $a = (T, E, K)$. For this sequence, $g^a_{(1,T)} = 1$, $g^a_{(2,E)} = 1$, $g^a_{(3,K)} = 1$, and all other $g_i^a = 0$.

Following Eq. (1), the probability of a sequence of K genotype frequency $(\boldsymbol{z}(t_k))_{k=1}^{K} = (\boldsymbol{z}(t_1), \boldsymbol{z}(t_2), \ldots, \boldsymbol{z}(t_K))$, conditioned on an initial distribution of genotype frequencies $\boldsymbol{z}(t_0)$, is given by the product of the individual transition probabilities,

$$P((\boldsymbol{z}(t_k))_{k=0}^{K}) = \prod_{k=0}^{K-1} P\left(\boldsymbol{z}(t_{k+1}) \mid \boldsymbol{z}(t_k)\right) P(\boldsymbol{z}(t_0)). \quad \textbf{(4)}$$

Inferring fitness effects of mutations with popDMS

We view sequencing results in a DMS experiment as measurements of the genotype frequency vectors $\boldsymbol{z}(t)$. To infer the functional effects of mutations, we apply Bayes' theorem, seeking the selection coefficients $\boldsymbol{s}$ that maximize the posterior probability of the entire evolutionary trajectory Eq. (4). This includes a Gaussian prior distribution for the selection coefficients

$$P_{\text{prior}}(s_i) \propto e^{-\gamma s_i^2/2}. \quad \textbf{(5)}$$

Here γ encodes of the width of the prior distribution, which can also be thought of as L_2-norm regularization of the selection coefficients. Since we optimize γ based on the data, our inference framework is not Bayesian in the strict sense, but it is effectively maximum likelihood inference with ridge regression. This prior will act to shrink the estimates of all mutation effects (including the wild-type) toward zero, so that large selection coefficients are not inferred without strong evidence.

The overall posterior distribution for the selection coefficients is then given by

$$P_{\text{post}}(\boldsymbol{s}) \propto \mathcal{L}\left((\boldsymbol{z}(t_k))_{k=1}^{K}\right) \prod_i P_{\text{prior}}(s_i), \quad \textbf{(6)}$$

where the likelihood of the data $\mathcal{L}$ is given by Eq. (4).

Following recent computational advances[2], to simplify the likelihood, we consider the diffusion limit of the WF model. In this limit, we assume N is large and the $\boldsymbol{s}$ and μ_{ab} are small, i.e., formally of order $O(1/N)$. Then we derive the Fokker-Plank (FP) equation[1,3,4] from the WF process Eq. (4), which describes the evolution of the probability density of genotype frequencies,

$$\frac{\partial p(\boldsymbol{z},t)}{\partial t} = \sum_a \frac{\partial}{\partial z_a}\left(\sum_b \frac{\partial}{\partial z_b}\frac{C_{ab}(\boldsymbol{z})}{2N} - d_a(\boldsymbol{z})\right) p(\boldsymbol{z},t). \quad \textbf{(7)}$$

Here $(d_a(\boldsymbol{z}))_a$ is referred to as the drift and $(C_{ab}(\boldsymbol{z}))_{ab}$ is the diffusion. Note that there are some differences in terminology between population genetics and FP equations. The "drift" term in the Fokker-Planck equation does *not* describe genetic drift in population genetics; instead, genetic drift is captured by the "diffusion" term.

The drift and diffusion terms arise from the first and second order cumulants of the binomial process Eq. (1), respectively:

$$\int d\boldsymbol{z}'(z'_a - z_a) P(\boldsymbol{z}' \mid \boldsymbol{z}) = d_a(\boldsymbol{z}) + O(1/N^2)$$

$$\int d\boldsymbol{z}'(z'_a - z_a)\left(z'_b - z_b\right) P(\boldsymbol{z}' \mid \boldsymbol{z}) = C_{ab}(\boldsymbol{z})/N + O(1/N^2). \quad \textbf{(8)}$$

Drift is characterized by selection pressure and mutation effects. By taking the leading orders in $O(1/N)$, then drift and diffusion terms can be expressed as:

$$
\begin{aligned}
d_a(\boldsymbol{z}) &= C_{aa}(\boldsymbol{z})s_a + \sum_{b\neq a} C_{ab}(\boldsymbol{z})s_b + \mu_a^{\text{fl}} \\
\mu_a^{\text{fl}} &= \sum_b (\mu_{ba}z_b - \mu_{ab}z_a) \\
C_{ab}(\boldsymbol{z}) &= \begin{cases} z_a(1-z_a)/N & \text{for } a=b \\ -z_a z_b/N & \text{for } a\neq b \end{cases}
\end{aligned}
\tag{9}
$$

Here, μ_a^{fl} is a net flux consisting of incoming probability flux from all genotypes b to a and outgoing flux from genotype a to all other genotypes b (due to mutation). We can then convert the FP equation Eq. (7) with drift and diffusion Eq. (9) into an expression that describes the probabilities of genotype frequency trajectories,

$$
\begin{aligned}
&P(\boldsymbol{z}(t_{k+1}) \mid \boldsymbol{z}(t_k)) \propto \exp\left(-N\mathcal{S}(\boldsymbol{z}(t_{k+1}) \mid \boldsymbol{z}(t_k))\right) \\
&\mathcal{S}(\boldsymbol{z}(t_{k+1}) \mid \boldsymbol{z}(t_k)) = \frac{1}{2\Delta t_k}(\boldsymbol{\Delta z}(t_k) - \Delta t_k \boldsymbol{d}(\boldsymbol{z}(t_k)))^\top \\
&\qquad \times C(\boldsymbol{z}(t_k))^{-1}(\boldsymbol{\Delta z}(t_k) - \Delta t_k \boldsymbol{d}(\boldsymbol{z}(t_k)))\,.
\end{aligned}
\tag{10}
$$

Here, we denote $\boldsymbol{\Delta z}(t_k)$ as frequency change $\boldsymbol{z}(t_{k+1}) - \boldsymbol{z}(t_k)$. The last expression enables us to obtain an analytical solution for the optimal selection that maximizes the posterior distribution over the evolution Eq. (6).

Finally, the selection coefficients that maximize Eq. (6) are given by

$$
\hat{s}_i = \sum_j \left[\sum_{k=0}^{K-1} \Delta t_k C(t_k) + \gamma I/N\right]^{-1}_{ij} \left(\Delta x_j - \mu_j^{\text{fl}}\right),
\tag{11}
$$

where $\Delta t_k = t_{k+1} - t_k$, $\Delta x_j = x_j(t_K) - x_j(t_0)$, and μ_j^{fl} is the net expected change in the frequency of variant j over the course of the experiment due to mutation alone. Typically, μ_{fl} is assumed to be zero, except for experiments involving viral replication, where mutation rates can be high enough to produce observable changes in frequency. Here $C(t)$ is the covariance matrix of variant frequencies $x_i(t) = \sum_{a=1}^{M} g_i^a z_a(t)$, which has entries

$$
C_{ij}(t) = \begin{cases} x_i(t)(1-x_i(t)) & i=j \\ x_{ij}(t) - x_i(t)x_j(t) & i\neq j\,. \end{cases}
\tag{12}
$$

Here $x_{ij}(t) = \sum_{a=1}^{M} g_i^a g_j^a z_a(t)$ is the frequency of genotypes at time t that contain both variants i and j.

The estimate of the selection coefficients $\hat{s}_i$ given in Eq. (11) can be explained intuitively. First, for simplicity, consider the matrices $C(t_k)$ to be diagonal. Then, the estimate for $\hat{s}_i$ depends on how much variant i has increased in frequency over the course of the experiment, after correcting for changes in frequency that are not due to functional selection, $(\Delta x_i - \mu_i^{\text{fl}})$. This quantity is normalized by the variance of the variant frequency $x_i(t_k)$ over time (Eq. (12)). In the limit that $x_i(t_k)$ is small (and again, that the off-diagonal terms are zero), the estimate for $\hat{s}_i$ is similar to an enrichment ratio, because in this limit $1 - x_i(t_k) \approx 1$. However, this estimate is also shrunk by a factor of γ due to the prior distribution for the selection coefficients. Importantly, the variance also becomes small when $x_i(t_k)$ is close to one, as is often the case for wild-type (WT) or reference amino acids in DMS experiments. This distinguishes the treatment of WT variants in popDMS as compared to ratio-based methods and regression-based methods that do not assume logistic growth.

Off-diagonal terms in Eq. (11) account for the influence of genetic background on changes in variant frequency. For example, a variant i may increase in frequency not because it has a beneficial functional effect, but rather because it appears on the same genetic sequence with other beneficial variants more often than expected by chance (i.e., positively covarying with other beneficial variants; see Eq. (12)). In population genetics, this phenomenon is referred to as genetic hitchhiking[5]. In DMS data, covariances cannot always be computed due to limited read lengths, but this information can be used to enhance predictions when it is available.

To derive Eq. (11), we assumed that the number of individuals in the population, N, is constant. However, in experiments (and in real populations), N can vary in time. Incorporating time-varying population sizes leads to similar estimates of selection, but with a larger uncertainty in the inferred parameters (see ref.[6] for a related model). For simplicity, we will maintain the assumption that N is constant. Additionally, in the discussion below we will absorb the population size N into the definition of γ, so that the strength of the regularization does not rely on an arbitrary definition of population size.

Joint estimates of selection coefficients across experimental replicates

We model experimental replicates as alternative evolutionary histories, subject to the same functional effects of mutations but with different stochastic realizations of evolution (and potentially different starting conditions). The posterior probability for the selection coefficients across R replicates is then given by

$$
P_{\text{post}}(\boldsymbol{s}) \propto \prod_{r=1}^{R} \mathcal{L}\left((\boldsymbol{z}^r(t_k))_{k=1}^{K_r}\right) \prod_i P_{\text{prior}}(s_i)\,.
\tag{13}
$$

Here each experimental replicate has a different index r, and the likelihood across all replicates is the product of the likelihood for each individual replicate. Since each $\mathcal{L}$ is Gaussian in the selection coefficients, the product is also Gaussian, and the MAP selection coefficients can be computed as in Eq. (11), yielding

$$
\begin{aligned}
\hat{s}_i = \sum_j &\left[\sum_{r=1}^{R}\sum_{k=0}^{K-1} \Delta t_k C^r(t_k) + \gamma I/N\right]^{-1}_{ij} \\
&\times \sum_{r=1}^{R}\left(\Delta x_j^r - \mu_j^{r,\text{fl}}\right).
\end{aligned}
\tag{14}
$$

Correction for sequencing errors

For some data sets, information on sequencing error rates is available. For example, this can be obtained by sequencing a library consisting of all WT sequences, so that all differences from WT are likely attributable to sequencing errors. When this data is available, we compute corrected mutant and WT counts by subtracting the expected contributions from sequencing errors.

Optimizing the regularization strength

For simplicity, we incorporate the WF population size N into the prior parameter γ to define an effective regularization strength $\gamma' = \gamma/N$. Larger values of γ' put a higher penalty on inferred selection coefficients, thereby suppressing their values, but also limiting the effects of sampling noise. Smaller values of γ' allow for the inference of larger selection coefficients, but in turn, these estimates are more sensitive to noise.

One can choose a single value of γ' to use for all data sets, but this parameter can also easily be optimized for an individual data set. The most computationally intensive step in inferring mutation effects (i.e., selection coefficients) with popDMS is computing the variant frequencies and covariances from sequencing data. After this step has been completed, it is straightforward to sweep through a range of γ' values and test their results for each data set.

We found that the average correlation of inferred mutation effects between replicates typically behaves like a logistic function of $\log(\gamma')$. For very small values of γ', sampling noise is not effectively suppressed, and the correlation of inferred mutation effects between replicates is lower. As γ' increases, noise is suppressed, leading to higher correlations between replicates. At high values of γ', high correlations between replicates are typically preserved, but the inferred selection coefficients are shrunk strongly towards zero.

We reasoned that an optimal choice for the regularization strength γ' would be the smallest value of γ' that effectively suppresses sampling noise, as this would avoid shrinking estimated selection coefficients unnecessarily. To compute this value, for each experimental data set described below, we swept through values of γ' in even logarithmically spaced steps from roughly $1/B$, where B is the maximum read depth, to 1000. For each value of γ', we computed the correlation between replicates. We then computed the difference $\Delta R = R_{\text{max}} - R_{\text{min}}$ between the maximum correlation and minimum correlation between replicates across all values of γ'. To determine the optimum value of γ', we started with the value that corresponds to the maximum correlation between replicates. We then identified the γ' where R values drop the most significantly. If R does not decrease more than a threshold, we used the smallest γ value where R decreases by 10% of R_{max}.

While sweeping through values of γ' improves our consistency across data sets, allowing us to adjust our regularization to match the level of noise in the data, we emphasize that this step is not essential to obtain robust results. A simple choice of $\gamma' = 0.1$ is nearly optimal for every data set we considered, with the exception of the influenza PR8 study[7]. This data set is the only one in which the correlation between replicates is not roughly a logistic function of the regularization strength.

Generating logo plots with popDMS

Inferences from DMS data such as amino acid preferences (derived from enrichment ratios) have often been used to generate logo plots that show the relative dominance of different amino acids at each site. However, while preferences naturally sum to one, selection coefficients inferred by popDMS can be both positive and negative. To obtain preference-like logo plots using selection coefficients inferred by popDMS, computed exponentially transformed values

$$p_i = e^{\beta s_i}\,, \tag{15}$$

where the scaling factor β was approximately chosen to maximize the correlation between the transformed selection coefficients p_i and amino acid preferences for the same data set.

Inference of epistasis

We extended our approach to infer pairwise epistatic interactions between variants by adding epistatic interactions s_{ij} to the previous fitness function Eq. (3), i.e.,

$$f_a = 1 + \sum_i s_i g_i^a + \sum_i \sum_{j \neq i} s_{ij} g_i^a g_j^a\,. \tag{16}$$

As for the selection coefficients defined above, if an epistatic interaction s_{ij} is positive, then the presence of variants i and j together increases fitness more than would be expected from the combined effect of the individual variants. When s_{ij} is negative, variants i and j together are more deleterious than expected if they were independent.

With this extension of the fitness model, one can then compute the posterior probability for the change in genotype frequencies, as in Eq. (6). We also assume a Gaussian prior distribution for the epistatic interactions that is centered at zero and has the same width as for the selection coefficients. The MAP selection coefficients for the selection coefficients and epistatic interactions have a form analogous to Eq. (11), but with an expanded index that runs over all variants i and all *pairs* of variants (i,j). Additional terms in the covariance matrix are then given by

$$\begin{aligned} C_{i,(i,j)}(t) &= x_{ij}(t)\,(1 - x_i(t))\,, \\ C_{i,(j,k)}(t) &= x_{ijk}(t) - x_i(t)x_{ij}(t)\,, \\ C_{(i,j),(i,j)}(t) &= x_{ij}(t)\,(1 - x_{ij}(t))\,, \\ C_{(i,j),(i,k)}(t) &= x_{ijk}(t) - x_{ij}(t)x_{ik}(t)\,, \\ C_{(i,j),(k,l)}(t) &= x_{ijkl}(t) - x_{ij}(t)x_{kl}(t)\,, \end{aligned} \tag{17}$$

with

$$\begin{aligned} x_{ijk}(t) &= \sum_{a=1}^{M} g_i^a g_j^a g_k^a z_a(t)\,, \\ x_{ijkl}(t) &= \sum_{a=1}^{M} g_i^a g_j^a g_k^a g_l^a z_a(t)\,. \end{aligned} \tag{18}$$

popDMS differs from some alternatives to estimating epistasis in that information about pairwise interactions is gained from all sequences that bear two or more non-reference variants. For example, one previously developed approach effectively estimated the fitness of sequences with exactly two mutations and compared this with estimates of the fitness for corresponding single mutants to estimate the strength of epistatic interaction between the mutations[8].

At present, inferring epistatic interactions from DMS data with popDMS is only computationally feasible for short sequences due to the large size of the covariance matrix. Alternative approaches that strictly enforce sparsity and reduce the number of possible interactions to estimate could potentially ease these computational restrictions.

Testing performance in simulations

We simulated evolution following the WF model over a number of generations to test the performance of popDMS. To reproduce finite sampling statistics similar to those observed in experimental data, we used the initial genotype frequency data from an experimental data set[9]. We ordered the variants by frequency at each site and inferred a best-fit multinomial model describing the frequency distribution across sites using PyStan[10]. This inferred distribution thus captures a typical hierarchy of frequencies observed in DMS experiments, from high frequency (WT/reference) variants to rare ones, whose counts may be of the same order as the read depth.

In our simulations, selection coefficients for all variants were chosen at random from a normal distribution with mean zero and standard deviation 0.1. True starting frequencies were sampled at random from the inferred multinomial distribution using PyStan. We then simulated up to 10 generations of evolution following the WF model, here assuming a mutation rate of zero and population size of $N = 10^8$. From these true trajectories, we obtained finitely sampled frequency trajectories by multinomial sampling from the true frequencies at each generation, with various choices for the sampling depth. To highlight stochasticity, we used a sampling depth of $B = 5 \times 10^4$ sequences in **Supplementary Fig. 1a**.

We used this data to compute the average correlation for selection coefficients inferred from different replicates using popDMS, which varies depending on the number of generations of data used (**Supplementary Fig. 1b**). Intuitively, observing the evolution for a longer time leads to more precise estimates.

We compared the results of popDMS against other common approaches, which we implemented as described below. To compute enrichment ratios, we compare the fraction of reads with a particular variant a pre- and post-selection,

$$E_a = \frac{n^a_{\text{post}}/B_{\text{post}}}{n^a_{\text{pre}}/B_{\text{pre}}} \,. \tag{19}$$

Here n^a_{pre} and n^a_{post} are number of reads with variant a before selection and after selection, respectively. Similarly, B_{pre} and B_{post} represent the total number of reads before and after selection. To compute log ratio scores, we used the natural logarithm of the enrichment ratios,

$$E^{\log}_a = \log\left(\frac{n^a_{\text{post}}/B_{\text{post}}}{n^a_{\text{pre}}/B_{\text{pre}}}\right) . \tag{20}$$

Finally, log ratio regression scores were computed by calculating the logarithm of the enrichment ratio Eq. (20) for each variant at each generation, then extracting the slope of the linear model the best fits the change in log enrichment ratios over time.

Effects of strong noise on read counts

We further extended the simulations described above to model the effects of strong noise in read counts, which can appear in DMS experiments[11,12]. We modeled additional sampling noise for read counts using a negative binomial distribution $P_{\text{NB}}(\lambda, r)$, a heavy-tailed distribution that has been used in prior work to model overdispersion in sequence count distributions. Here, λ and r are the mean (expected) read counts and the dispersion parameter, respectively. We used dispersion parameters derived from the analysis of experimental data: $\lambda_a = Bn_a/N$ and $r = r(\lambda) = \beta\lambda^\alpha$ with $\alpha = 0.69$ and $\beta = 0.8$ (ref.[13]). In the expression for λ_a, B is the sample size (i.e., the total number of reads) and N is the total population size. Subsequently, we obtained an ensemble of read counts sampled from the WF model simulations with the fitness function described above.

We then compared the inferred selection coefficients with the true ones (**Supplementary Fig. 2**), using the same procedure as for experimental data sets. We found that the correlation obtained by popDMS (Pearson's $R = 0.94$) exceeded ones using log-preferences, log-ratio regression, or log-enrichment ratios (Pearson's $R = 0.65$, $R = 0.53$, and $R = 0.53$, respectively; **Supplementary Fig. 2**). For the regularization strength, we used the same procedures as for experimental data. We used three replicates for all inference methods.

DMS data sets

Data sets used in this paper were obtained from 17 publications[7–9,14–27]. Additional information about these data sets, and the methods used to analyze them, is summarized in **Table 1**.

Comparison with prior studies of epistasis

Here we analyzed a data set from Araya and collaborators, which explored epistasis in the WW domain of the hYAP65 protein[8]. There, they define epistasis in a way that differs from our definition (i.e., the s_{ij} in Eq. (16)). For each genotype variant a, Araya et al. define a parameter $W_a = 2^{(S_a - S_{\text{WT}})}$, where the S_a are best-fit slopes of the logarithmic enrichment ratios for variant a. S_{WT} is the slope for the WT variant, which they use to normalize the results. They use the quantity $\epsilon_{ab} = W_{ab} - W_a W_b$ as the primary metric of epistasis. Here, a and b represent genotypes with a single mutation, and ab the genotype that features only these two mutations.

When the frequency of a variant is small, the S_a computed by Araya et al. are similar to our f_a. Thus, to compare the quantities inferred by Araya et al. to our s_{ij}, we computed a set of transformed scores, which we write as

$$\tilde{s}_{ij} = \log_2(W_{ij}) - \log_2(W_i)\log_2(W_j)\ . \tag{21}$$

There is good overall agreement in the epistatic interactions s_{ij} inferred by popDMS and the transformed interactions $\tilde{s}_{ij}$, computed from the W values of Araya et al. (Pearson's $R = 0.73$, Spearman's $\rho = 0.75$). **Figure 2a** similarly shows broad agreement between the sum of squared epistatic interactions between variants at each pair of sites in the WW domain, though those inferred by popDMS are sparser (**Figure 2b**).

Comparison with natural frequencies of influenza variants

In general, it is challenging to validate inferences about the fitness or functional effects of amino acid variants inferred from DMS experiments because "ground truth" measurements for these effects do not exist. However, one possible method of validation is to compare the inferred fitness effects of variants to the frequency of mutations observed in natural populations. This approach was explored by Thyagarajan and collaborators in their study of the effects of mutations in the influenza hemagglutinin protein[21].

We performed a similar analysis to compare our results to fitness effects inferred using enrichment ratios for the same data set[21]. While it is possible to directly correlate variant frequency and the inferred fitness effect of the variant, this connection is not entirely natural because frequency should be determined not just by the fitness effect of a variant, but also by the relative fitness effects of other possible variants at the same site.

To make a clearer connection with the data, we reasoned that, in most cases, the amino acid with the highest frequency in natural populations should be the variant with the highest fitness at each site. We thus ranked the fitness effects of each amino acid variant at the same site, and computed the rank of the top variant according to both selection coefficients inferred by popDMS and enrichment ratios. For popDMS, the amino acid most frequently observed in natural populations had an average rank of 2.1 across sites (median 1), compared to an average rank of 2.7 (median 1) for enrichment ratios.

To determine the extent to which the amino acid that is most frequently observed in natural populations is predicted to be dominant at each site, we also computed a z score for the most frequent variant at each site. This was computed by taking the metric of fitness (selection coefficients or enrichment ratios) for the most frequent variant at each site, subtracting the mean value for the same site, and dividing by the standard deviation of values at that site. We found an average z score for the most frequent variant of 3.5 using popDMS, compared to 2.6 for enrichment ratios.

Thus, we find that selection coefficients match well with the corresponding frequencies of amino acid variants in a natural population. Results obtained using popDMS also compare favorably with prior results computed using enrichment ratios[21].

Data and code

Raw data and code used in our analysis are available in the GitHub repository located at `https://github.com/bartonlab/paper-DMS-inference`. This repository also contains Jupyter notebooks that can be run to reproduce the results presented here. Code for popDMS alone, without the analysis contained in this paper, is also provided in a separate GitHub repository at `https://github.com/bartonlab/popDMS`. popDMS is coded in Python3 and C++.

References

1. Ewens, W. J. *Mathematical population genetics: theoretical introduction*, vol. 27 (Springer, 2004).
2. Sohail, M. S., Louie, R. H., McKay, M. R. & Barton, J. P. MPL resolves genetic linkage in fitness inference from complex evolutionary histories. *Nature Biotechnology* **39** (2021).
3. Kimura, M. Diffusion models in population genetics. *Journal of Applied Probability* **1**, 177–232 (1964).
4. Tataru, P., Bataillon, T. & Hobolth, A. Inference under a wright-fisher model using an accurate beta approximation. *Genetics* **201**, 1133–1141 (2015).
5. Smith, J. M. & Haigh, J. The hitch-hiking effect of a favourable gene. *Genetics Research* **23**, 23–35 (1974).
6. Lee, B. *et al.* Inferring effects of mutations on sars-cov-2 transmission from genomic surveillance data. *medRxiv* 2021–12 (2022).
7. Doud, M. B., Ashenberg, O. & Bloom, J. D. Site-specific amino acid preferences are mostly conserved in two closely related protein homologs. *Molecular Biology and Evolution* **32** (2015).
8. Araya, C. L. *et al.* A fundamental protein property, thermodynamic stability, revealed solely from large-scale measurements of protein function. *Proceedings of the National Academy of Sciences of the United States of America* **109** (2012).
9. Haddox, H. K., Dingens, A. S., Hilton, S. K., Overbaugh, J. & Bloom, J. D. Mapping mutational effects along the evolutionary landscape of HIV envelope. *eLife* **7** (2018).
10. Carpenter, B. *et al.* Stan: A probabilistic programming language. *Journal of statistical software* **76** (2017).
11. Kebschull, J. M. & Zador, A. M. Sources of pcr-induced distortions in high-throughput sequencing data sets. *Nucleic acids research* **43**, e143–e143 (2015).
12. Potapov, V. & Ong, J. L. Examining sources of error in pcr by single-molecule sequencing. *PloS one* **12**, e0169774 (2017).
13. Nemoto, T. *et al.* Acides: on-line monitoring of forward genetic screens for protein engineering. *Nature Communications* **14**, 8504 (2023).
14. Sourisseau, M. *et al.* Deep mutational scanning comprehensively maps how zika envelope protein mutations affect viral growth and antibody escape. *Journal of Virology* **93**, 10.1128/jvi.01291–19 (2019).
15. Roop, J. I., Cassidy, N. A., Dingens, A. S., Bloom, J. D. & Overbaugh, J. Identification of HIV-1 envelope mutations that enhance entry using macaque CD4 and CCR5. *Viruses* **12** (2020).
16. Dingens, A. S. *et al.* Complete functional mapping of infection- and vaccine-elicited antibodies against the fusion peptide of HIV. *PLoS Pathogens* **14** (2018).
17. Lee, J. M. *et al.* Deep mutational scanning of hemagglutinin helps predict evolutionary fates of human h3n2 influenza variants. *Proceedings of the National Academy of Sciences* **115**, E8276–E8285 (2018).
18. Soh, Y. S., Moncla, L. H., Eguia, R., Bedford, T. & Bloom, J. D. Comprehensive mapping of adaptation of the avian influenza polymerase protein PB2 to humans. *eLife* **8** (2019).
19. Hom, N., Gentles, L., Bloom, J. D. & Lee, K. K. Deep Mutational Scan of the Highly Conserved Influenza A Virus M1 Matrix Protein Reveals Substantial Intrinsic Mutational Tolerance. *Journal of Virology* **93** (2019).
20. Ashenberg, O., Padmakumar, J., Doud, M. B. & Bloom, J. D. Deep mutational scanning identifies sites in influenza nucleoprotein that affect viral inhibition by mxa. *PLoS Pathogens* **13** (2017).
21. Thyagarajan, B. & Bloom, J. D. The inherent mutational tolerance and antigenic evolvability of influenza hemagglutinin. *elife* **3**, e03300 (2014).
22. Starita, L. M. *et al.* Activity-enhancing mutations in an E3 ubiquitin ligase identified by high-throughput mutagenesis. *Proceedings of the National Academy of Sciences of the United States of America* **110** (2013).
23. Starita, L. M. *et al.* Massively parallel functional analysis of BRCA1 RING domain variants. *Genetics* **200** (2015).
24. Bridgford, J. L. *et al.* Novel drivers and modifiers of MPL-dependent oncogenic transformation identified by deep mutational scanning. *Blood* **135** (2020).
25. Findlay, G. M., Boyle, E. A., Hause, R. J., Klein, J. C. & Shendure, J. Saturation editing of genomic regions by multiplex homology-directed repair. *Nature* **513** (2014).
26. Starr, T. N. *et al.* Deep mutational scanning of sars-cov-2 receptor binding

domain reveals constraints on folding and ace2 binding. *cell* **182**, 1295–1310 (2020).
27. Lei, R. *et al.* Mutational fitness landscape of human influenza h3n2 neuraminidase. *Cell reports* **42** (2023).

	Protein	Source	Inference method (software)	Number of time points	Number of replicates	Run time (seconds)	Reference
1	Zika Virus Envelope	Virus	Enrichment ratio (dms_tools2)	2	3	10	Sourisseau et al., 2019 [14]
2	HIV-1 Envelope - BG505			2	3	13	Haddox et al., 2018 [9]
3	HIV-1 Envelope - BF520			2	3	12	
4	HIV-1 Envelope - BF520 - Human			2	2	8	Roop et al., 2020 [15]
5	HIV-1 Envelope - BF520 - Rhesus			2	2	8	
6	HIV-1 Envelope - BG505 - VRC34			2	2	9	Dingens et al., 2018 [16]
7	HIV-1 Envelope - BG505 - FP16			2	2	8	
8	HIV-1 Envelope - BG505 - FP20			2	2	8	
9	H3N2 Influenza Hemagglutinin Perth2009			2	4	14	Lee et al., 2018 [17]
10	H1N1 Influenza Polymerase Basic 2 - CCL141			2	3	14	Soh et al., 2019 [18]
11	H1N1 Influenza Polymerase Basic 2 - A549			2	3	14	
12	H1N1 Influenza Matrix Protein M1			2	3	5	Hom et al., 2019 [19]
13	H3N2 Influenza Nucleoprotein MxA		Enrichment ratio (dms_tools)	2	2	6	Ashenberg et al., 2017 [20]
14	H3N2 Influenza Nucleoprotein MxAneg			2	2	6	
15	H3N2 Influenza Nucleoprotein MS			2	2	6	
16	H1N1 Influenza Nucleoprotein PR8			2	3	9	Doud et al., 2015 [7]
17	H3N2 Influenza Nucleoprotein Aichi68C			2	2	6	
18	H1N1 Influenza Hemagglutinin WSN		Enrichment ratio (mapmuts)	2	3	11	Thyagarajan et al., 2014 [21]
19	H3N2 Influenza Neuraminidase NA		Enrichment ratio	2	2	6	Lei et al., 2023 [27]
20	SARS-CoV-2 Receptor Binding Domain Spike		Global epistasis	2	2	748	Starr et al., 2020 [26]
21	Ubiquitination factor E4B - Ube4b	Mouse	Enrichment ratio (Enrich)	4	2	266	Starita et al., 2013 [22]
22	BRCA1 RING Domain - Y2H 1	Human		4	3	2607	Starita et al., 2015 [23]
23	BRCA1 RING Domain - Y2H 2			4	3	2588	
24	BRCA1 RING Domain - E3			6	6	8253	
25	Myeloproliferative Leukemia Protein		Log ratio regression (Enrich2)	2	6	26	Bridgford et al., 2020 [24]
26	Myeloproliferative Leukemia Protein S505N			2	6	25	
27	hYAP65 WW domain - WW		Log ratio regression	4	2	31	Araya et al., 2012 [8]
28	BRCA1 exon 18 - DBR1			4	2	1237	Findlay et al., 2014 [25]

Supplementary Table 1. Summary of data sets studied in this work.

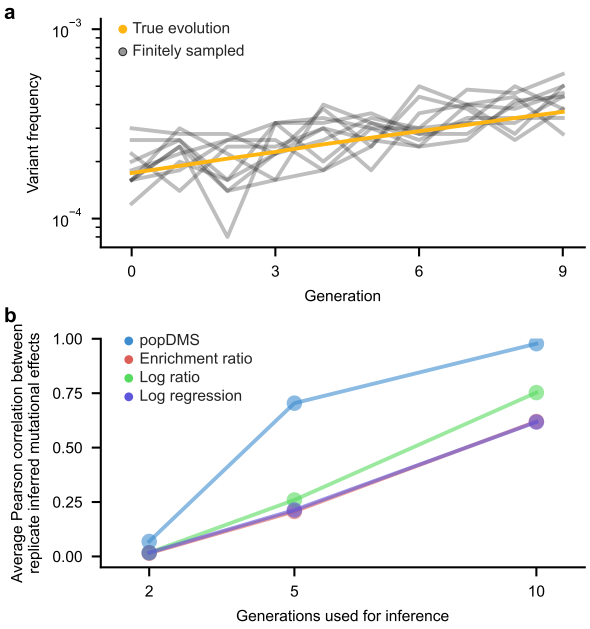

Supplementary Fig. 1. popDMS is robust to finite sampling error. **a**, Due to finite sampling of the data, variant frequencies can appear to fluctuate over time even if the underlying behavior is smooth, complicating inference. Results from an example simulation (Methods). **b**, As the number of generations used for inference in simulations increases, all methods become more robust. popDMS is especially robust in inferring mutation effects from limited data with few rounds of selection.

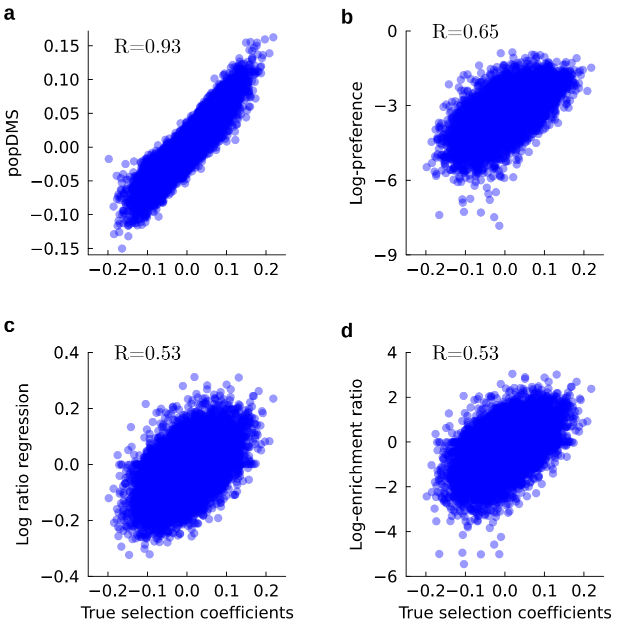

Supplementary Fig. 2. Comparison between true and inferred fitness effects of mutations in simulations including overdispersion of variant counts. a, Selection coefficients inferred via popDMS from simulations compared with the underlying true ones (see Methods). Correlations between true and inferred coefficients are higher for popDMS than for alternatives, including (**b**) log preferences, (**c**) log ratio regression, and (**d**) log enrichment ratios.

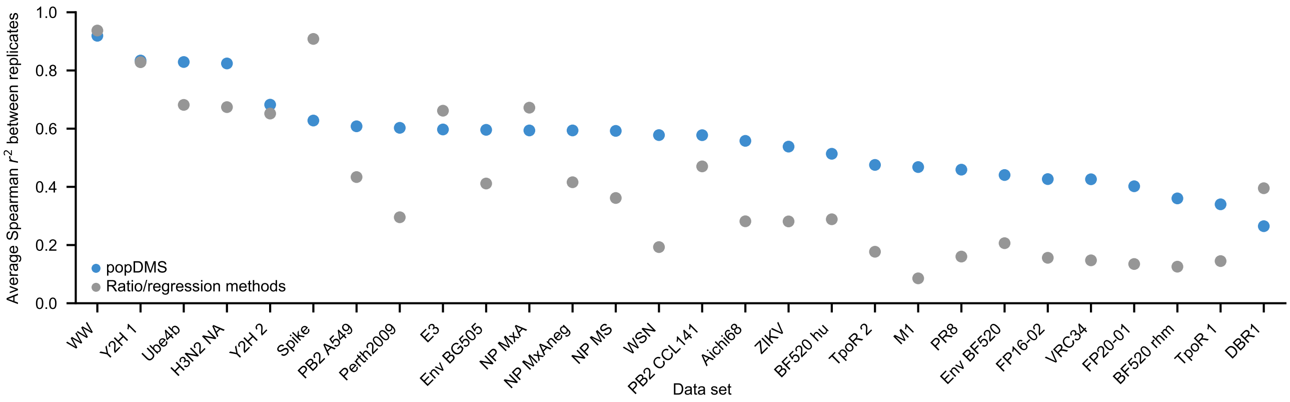

Supplementary Fig. 3. popDMS achieves robust rank correlations between mutation effects inferred from different experimental replicates. This figure is analogous to **Fig. 1b** in the main text, but plotting the Spearman r^2 between replicates instead of Pearson correlations.

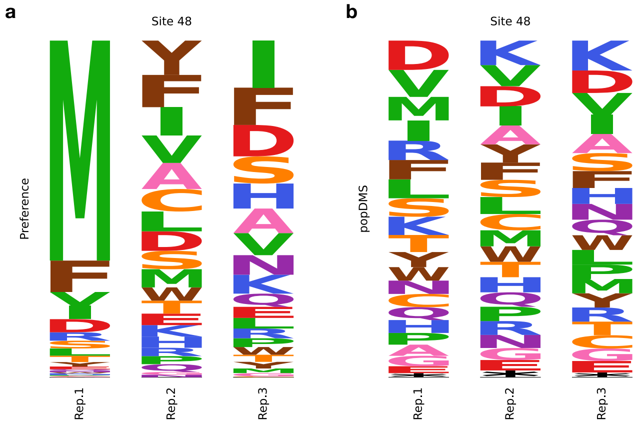

Supplementary Fig. 4. Typical example site where popDMS displays more consistent inferences between replicates. Here we examine preferences (**a**) and exponentially transformed selection coefficients (**b**) at site 48 in the HIV Env BG505 data set. popDMS values are more consistent across replicates. Unusually low counts for methionine in the initial library for the first replicate lead to a large enrichment ratio, which skews estimated mutational effects. The selection coefficient inferred for methionine from the first replicate alone is also enhanced, but more moderately so due to the influence of regularization and proportionality to the change in frequency Δx.

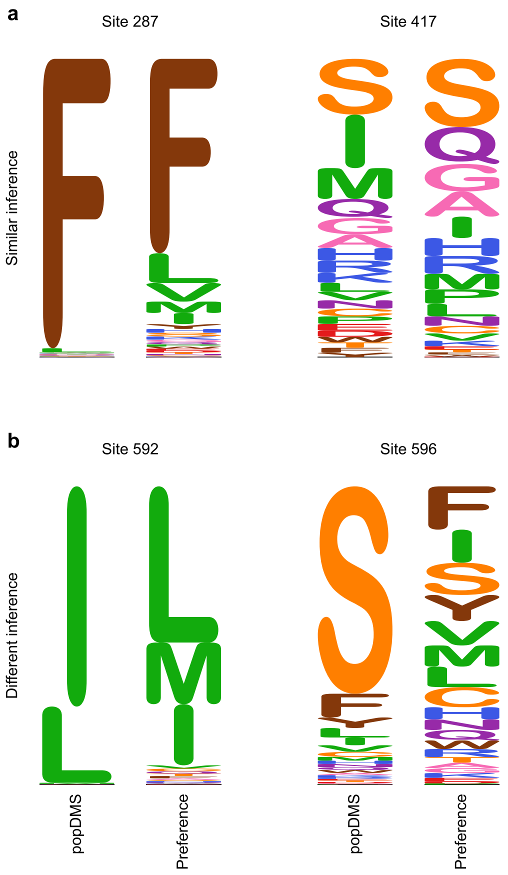

Supplementary Fig. 5. Comparison of selection at individual sites inferred by popDMS and enrichment ratios for the HIV-1 Env BG505 data set. **a**, Exponentially transformed selection coefficients inferred by popDMS (see Methods) are similar to preferences (normalized enrichment ratios) at sites 287 and 417. At site 287, both methods agree on the dominance of phenylalanine. At site 417, both methods find broad tolerance for different amino acid variants. **b**, In contrast, differences are observed between popDMS and preferences at sites 592 and 596. In both cases, popDMS finds the reference amino acid (isoleucine at site 592 and serine at site 596) to be strongly favored due to its increase in frequency during the experiment. These frequency changes were small relative to the initial frequency of the amino acid, but they were large considering the limited capacity for the amino acid to grow in frequency. This latter factor is captured by popDMS, but is not typically accounted for in ratio-based approaches.
